# Large-scale network analysis of the cerebrospinal fluid proteome identifies molecular signatures of frontotemporal lobar degeneration

**DOI:** 10.21203/rs.3.rs-4103685/v1

**Published:** 2024-03-28

**Authors:** Rowan Saloner, Adam Staffaroni, Eric Dammer, Erik C.B. Johnson, Emily Paolillo, Amy Wise, Hilary Heuer, Leah Forsberg, Argentina Lario Lago, Julia Webb, Jacob Vogel, Alexander Santillo, Oskar Hansson, Joel Kramer, Bruce Miller, Jingyao Li, Joseph Loureiro, Rajeev Sivasankaran, Kathleen Worringer, Nicholas Seyfried, Jennifer Yokoyama, William Seeley, Salvatore Spina, Lea Grinberg, Lawren VandeVrede, Peter Ljubenkov, Ece Bayram, Andrea Bozoki, Danielle Brushaber, Ciaran Considine, Gregory Day, Bradford Dickerson, Kimiko Domoto-Reilly, Kelley Faber, Douglas Galasko, Daniel Geschwind, Nupur Ghoshal, Neill Graff-Radford, Chadwick Hales, Lawrence Honig, Ging-Yuek Hsiung, Edward Huey, John Kornak, Walter Kremers, Maria Lapid, Suzee Lee, Irene Litvan, Corey McMillan, Mario Mendez, Toji Miyagawa, Alexander Pantelyat, Belen Pascual, Henry Paulson, Leonard Petrucelli, Peter Pressman, Eliana Ramos, Katya Rascovsky, Erik Roberson, Rodolfo Savica, Allison Snyder, A. Campbell Sullivan, Carmela Tartaglia, Marijne Vandebergh, Bradley Boeve, Howie Rosen, Julio Rojas, Adam Boxer, Kaitlin Casaletto

**Affiliations:** University of California, San Francisco; University of California, San Francisco; Emory University; Emory University School of Medicine; University of California, San Francisco; University of California, San Francisco; University of California, San Francisco; Mayo Clinic; University of California, San Francisco; University of California, San Francisco; Lund University; Lund University; Lund University; University of California, San Francisco; University of California San Francisco; Novartis Institutes for Biomedical Research, Inc.; Novartis Institutes for Biomedical Research; Novartis (United States); Emory University School of Medicine; UCSF; University of California at San Francisco; University of California, San Francisco; University of California at San Francisco; University of California, San Francisco; University of California, San Francisco; University of California, San Diego; University of North Carolina; Mayo Clinic; Vanderbilt University; Mayo Clinic in Florida; Harvard University; University of Washington; Indiana University; UC San Diego; UCLA Health System and David Geffen School of Medicine; Washington University; Mayo Clinic; Emory University; Columbia University; University of British Columbia; Columbia University; University of California, San Francisco; Mayo Clinic; Mayo Clinic; University of California, San Francisco; UCSD; Department of Neurology, University of Pennsylvania, Philadelphia, USA; Mayo Clinic; Johns Hopkins University; Houston Methodist; University of Michigan–Ann Arbor; Mayo Clinic; University of Colorado; University of California, Los Angeles; University of California, San Francisco; University of Alabama at Birmingham; Mayo Clinic; National Institutes of Health; University of Texas Health San Antonio; University of Toronto; University of Antwerp; Mayo Clinic; University of California, San Francisco; University of California, San Francisco; Memory and Aging Center, Department of Neurology, University of California, San Francisco; University of California, San Francisco

## Abstract

The pathophysiological mechanisms driving disease progression of frontotemporal lobar degeneration (FTLD) and corresponding biomarkers are not fully understood. We leveraged aptamer-based proteomics (> 4,000 proteins) to identify dysregulated communities of co-expressed cerebrospinal fluid proteins in 116 adults carrying autosomal dominant FTLD mutations (*C9orf72, GRN, MAPT*) compared to 39 noncarrier controls. Network analysis identified 31 protein co-expression modules. Proteomic signatures of genetic FTLD clinical severity included increased abundance of RNA splicing (particularly in *C9orf72* and *GRN*) and extracellular matrix (particularly in *MAPT*) modules, as well as decreased abundance of synaptic/neuronal and autophagy modules. The generalizability of genetic FTLD proteomic signatures was tested and confirmed in independent cohorts of 1) sporadic progressive supranuclear palsy-Richardson syndrome and 2) frontotemporal dementia spectrum syndromes. Network-based proteomics hold promise for identifying replicable molecular pathways in adults living with FTLD. ‘Hub’ proteins driving co-expression of affected modules warrant further attention as candidate biomarkers and therapeutic targets.

## Introduction

Frontotemporal lobar degeneration (FTLD) is among the most common causes of early age of onset dementia (< 65 years), resulting in progressive clinical syndromes that can feature behavioral, language, and/or motor deficits^1,2^. The majority of FTLD is comprised of two major molecular subtypes, defined by pathological accumulation and cellular inclusions of hyper-phosphorylated tau (FTLD-tau) or transactive response DNA-binding protein 43 kDa (FTLD-TDP)^3^. Current *in-vivo* molecular biomarkers to aid in diagnosis of sporadic forms of FTLD are limited to nonspecific indicators of neurodegeneration and axonal loss (e.g., neurofilament light chain [NfL]), as well as markers of Alzheimer’s disease (AD) pathology (e.g., Aβ_42/40_, P-tau181) to rule out AD. Although NfL predicts future clinical decline across FTLD clinical syndromes^4–6^, a single biomarker does not capture the biological complexity of FTLD and it remains unclear which biological pathways most strongly correlate with disease progression^7^. Molecular signatures that are detectable *in-vivo*, inform underlying pathophysiology, and contribute to clinical progression are urgently needed to advance understanding of FTLD pathogenesis and guide biomarker development, which will in turn enhance FTLD clinical trials through improved selection of patients and measurement of treatment effects^8^.

Underlying FTLD pathology can be confidently predicted in inherited cases caused by autosomal dominant mutations, which represent approximately 20–30% of all cases^9^. Observational study of FTLD mutation carriers has provided a unique opportunity to chart clinical and biomarker changes across presymptomatic and symptomatic stages of FTLD^5,8,10^. Autosomal dominant forms of FTLD-TDP are most often caused by mutations in chromosome 9 open reading frame 72 (*C9orf72* [*C9*]) or progranulin (*GRN*). The majority of autosomal dominant FTLD-tau is due to mutations in microtubule-associated protein tau (*MAPT*)^5,9^. Current understanding of the molecular mechanisms underlying autosomal dominant FTLD has been primarily ascertained from postmortem and non-human studies^3,11^. Hexanucleotide repeat expansions in the *C9orf72* gene may produce both loss-of-function (haploinsufficiency) and toxic gain-of-function (e.g., repeat RNA foci, dipeptide repeat protein aggregates) mechanisms that impair RNA metabolism, nucleocytoplasmic transport, and proteostasis pathways that contribute to nuclear depletion and cytoplasmic aggregation of TDP-43^12,13^. *GRN* mutations cause haploinsufficiency of progranulin, a lysosomal protein that regulates immune and autophagy pathways implicated in TDP-43 dyshomeostasis^14,15^. *MAPT* mutations alter tau binding to microtubules and promote tau aggregation, which directly compromises axonal transport, synaptic signaling, cellular metabolism, and the extracellular scaffolds that support neuronal structures^16–18^.

Recent large-scale proteomics studies in AD are contributing to discovery of key biological networks and individual proteins reflective of AD pathophysiology, which extend far beyond the classic hallmarks of misfolded amyloid and tau^19,20^. These studies have employed powerful systems biology approaches in brain tissue and biofluids (e.g., cerebrospinal fluid [CSF], blood) to identify communities of co-expressed proteins that are reliably altered in clinical cohorts that span the AD disease severity continuum^20–22^. Despite the potential to identify novel molecular signatures underlying disease progression, unbiased large-scale analysis of the biofluid proteome in living FTLD patients has lagged.

The present study employed a large-scale aptamer-based proteomic approach (SomaScan) to generate a CSF protein network in 116 participants with autosomal dominant FTLD mutations (*C9orf72, GRN, MAPT*). Network analysis identified dysregulated communities of CSF proteins (modules) involved in RNA splicing, protein degradation, extracellular matrix, and synaptic pathways. These modules were strongly correlated with cross-sectional and longitudinal markers of FTLD clinical progression. We validated the generalizability of our genetic FTLD protein network by demonstrating preservation of network module alterations in CSF of progressive supranuclear palsy (PSP), a sporadic 4-repeat FTLD tauopathy. We further evaluated the disease-specificity and biologic reproducibility of protein network associations by reconstructing modules in a third clinical cohort of sporadic and genetic FTLD and AD using an orthogonal proximity-extension assay based proteomic approach (Olink). Collectively, our approach and results provide new insights regarding *in-vivo* molecular signatures of central nervous system dysfunction that occur across the disease severity spectrum of FTLD.

## Results

### Participant Characteristics.

Supplementary Table 1 reports demographic and clinical characteristics for the 116 autosomal dominant FTLD mutation carriers and 39 familial controls, representing cognitively normal non-mutation carriers from families with a known *C9orf72, GRN*, or *MAPT* mutation. Consistent with the broader ALLFTD cohort^10^, *C9orf72* was the most common mutation (N = 47), followed by MAPT (N = 37) and *GRN* (N = 32). At baseline, almost half of all mutation carriers were presymptomatic (global CDR^®^+NACC-FTLD = 0; 54 [47%]; 24 [51%] *C9orf72*, 12 [39%] *GRN*, 18 [49%] MAPT). *GRN* carriers were on average older (mean age = 57.9 years) than *C9orf72* (mean age = 49.9 years), *MAPT* (mean age = 44.5 years), and controls (mean age = 45.8 years). Mutation carriers and controls were comparable in terms of sex (mutation carriers: 49–55% female vs. controls: 59% female) and education (mutation carriers: mean = 15.0 to 15.7 years vs. controls: mean = 15.6 years). The median [IQR] of annual study visits was 3 [1.75, 4] for mutation carriers and 3 [2, 5] for controls.

### Genetic FTLD Protein Co-expression Network.

**Figure 1:**
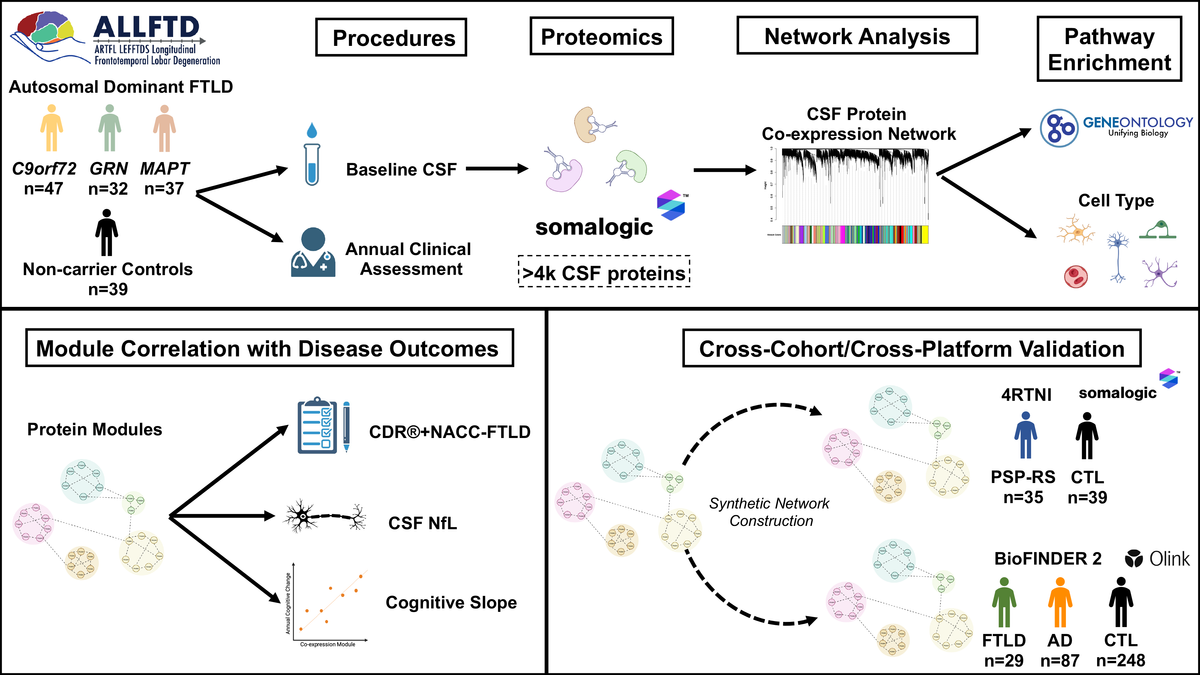
Study Overview. Cerebrospinal fluid (CSF) was collected in 116 carriers of autosomal dominant mutations for frontotemporal lobar degeneration (*FTLD; 47 C9orf72* [*C9*], 32 *GRN*, 37 *MAPT*) and 39 non-carrier controls with a family history of genetic FTLD. CSF was analyzed on a modified aptamer-based assay (SomaScan). After data processing, a total of 4,138 proteins were quantified. High-dimensional proteomic data were organized into modules of protein co-expression using weighted gene co-expression network analysis (WGCNA). CSF protein co-expression modules from the genetic FTLD network were functionally annotated using gene set and cell type enrichment approaches. CSF genetic FTLD modules were examined in relation to cross-sectional (CDR^®^+NACC-FTLD, CSF neurofilament light [NfL]) and longitudinal (global cognitive trajectories) indicators of disease severity. In cross-cohort and cross-platform validation analyses, genetic FTLD modules were reconstructed in independent cohorts of sporadic PSP-Richardson syndrome (PSP-RS) and controls (4RTNI; SomaScan) and FTLD, AD, and controls (BioFINDER 2; Olink).

**Figure 2:**
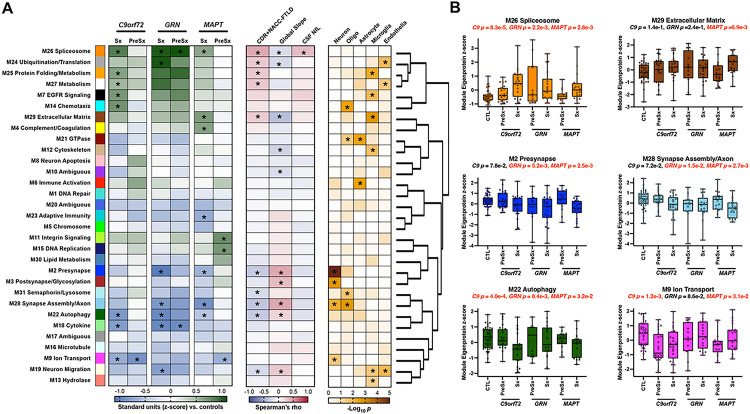
Genetic FTLD Protein Co-Expression Network. **A)** A cerebrospinal fluid (CSF) protein co-expression network was built using weighted gene correlational network analysis (WGCNA). The genetic FLTD network consisted of 31 protein co-expression modules. Module relatedness is shown in the dendrogram to the right. GO analysis was used to identify the principal biology represented by each module. Within genes, module eigenproteins in symptomatic (Sx) and presymptomatic (PreSx) variant carriers were compared against controls. Increased eigenprotein abundance in FTLD is indicated in green, whereas decreased eigenprotein abundance is indicated in blue. Module eigenproteins were correlated with disease outcomes, including CDR^®^+NACC-FTLD, global cognitive slope, and CSF NfL (red, positive correlation; blue, negative correlation). The cell type nature of each module was assessed by module protein overlap with cell-type-specific marker lists of neurons, oligodendrocytes, astrocytes, microglia and endothelia. Asterisks in the left heatmap indicate statistical significance after Tukey’s test. Asterisks in the middle and right heatmaps indicate statistical significance after false discovery rate correction. **B)** Module eigenprotein levels by case status for six of the most strongly FTLD-associated modules. Mutation carriers are grouped by Sx and PreSx individuals. *C*9: 24 PreSx, 23 Sx; GRN: 12 PreSx, 19 Sx; *MAPT*: 18 PreSx, 19 Sx. Differences in module eigenprotein by case status were assessed by one-way ANOVA with Tukey test. Gene-specific p-values represent the omnibus significance for gene-stratified comparisons vs. controls. Box plots represent the median and 25th and 75th percentiles, and box hinges represent the interquartile range of the two middle quartiles within a group. Min and max data points define the extent of whiskers (error bars). CTL, control.

The study design is presented in Fig. 1. Using weighted gene correlational network analysis (WGCNA), we built a protein co-expression network from 4,138 proteins across 155 CSF samples to identify protein communities that are dysregulated in FTLD (Fig. 2A). Network analysis revealed 31 protein co-expression modules across controls and mutation carriers. Module size ranged from 360 proteins (M1) to 48 proteins (M31). Protein module membership assignments are provided in Supplementary Table 2. Based on gene ontology and cell type enrichment analyses (Supplementary Table 3; Supplementary Fig. 1), 28 out of the 31 modules had a clear primary ontology that was used for annotation, while 3 modules did not evidence clear ontology (labeled “ambiguous”).

### CSF proteomic signatures of FTLD disease severity.

Analyses first combined FTLD mutation carriers across all 3 genes (N = 116) to maximize sample size. Module eigenproteins, calculated as the first principal component of module abundance, were compared across symptomatic mutation carriers, presymptomatic mutation carriers, and controls using one-way ANOVA with Tukey’s post-hoc correction, adjusting for age and sex. Module eigenproteins were also correlated with continuous indicators of functional severity (CDR^®^+NACC-FTLD sum of boxes), adjusting for p-values by false discovery rate (FDR) correction according to the Benjamini-Hochberg method. Seven of the 31 modules showed significant differences between symptomatic carriers and controls (Supplementary Table 4). Four of these modules exhibited particularly strong relationships with functional severity: M26 spliceosome, M2 presynapse, M28 synapse assembly/axon, and M22 autophagy. M26 spliceosome, highly enriched for proteins involved in mRNA splicing and nuclear transport, was markedly increased in symptomatic carriers vs. controls and positively correlated with functional severity (ρ = 0.41, FDR-*p* = 2.7e-6). M2 presynapse and M28 synapse assembly/axon, enriched for synaptic/neuronal (M2, M28) and oligodendrocyte (M28) cell-type markers, were decreased in symptomatic carriers vs. controls and negatively correlated with functional severity (M2: ρ=−0.33, FDR-*p* = 2.6e-4; M28: ρ=−0.35, FDR-*p* = 1.7e-4). M22 autophagy was also decreased in symptomatic carriers vs. controls and negatively correlated with functional severity (ρ=−0.33, FDR-*p* = 2.6e-4). CSF proteomic alterations were more subtle and less likely to survive post-hoc correction when comparing presymptomatic mutation carriers vs. controls. However, M9 ion transport, enriched for neuronal cell-type markers involved in ion channel activity and transport, was significantly decreased in presymptomatic carriers vs. controls.

We next examined how network modules associated with CSF NfL, an indicator of neuroaxonal degeneration, given that NfL is currently the most validated fluid biomarker for disease monitoring in genetic and sporadic FTLD^4,23,24^. Six modules associated with CSF NfL concentrations, measured by Simoa, at an unadjusted *p* < 0.05, with only M26 spliceosome surviving FDR-correction (ρ = 0.32, FDR-*p* = 9.3e-3). Upon closer examination, we observed that the SomaScan target for NfL (NEFL) was assigned to the M26 spliceosome module. However, NEFL was not a strong driver of M26 eigenprotein co-expression (intramodular kME = 0.32 [87th out of 88 module members]) and the relationship between M26 spliceosome and CSF NfL (Simoa) persisted when recalculating the M26 spliceosome eigenprotein without NEFL (ρ = 0.31, *p* = 9.1e-3).

### Gene-stratified FTLD proteomic signatures.

We performed gene-stratified analyses to determine whether proteomic signals observed in the full sample were driven by specific gene groups. For each gene group, we again compared module eigenprotein levels across symptomatic mutation carriers, presymptomatic mutation carriers, and controls using Tukey’s post-hoc correction. We also examined correlations with CDR^®^+NACC-FTLD sum of boxes and CSF NfL (Supplementary Table 4; Fig. 2B). FDR corrections were not applied to gene-stratified correlational analyses given the reduced sample sizes. Within each gene group, M26 spliceosome levels were increased and M22 autophagy module levels were decreased in symptomatic carriers vs. controls. However, symptomatic *C9orf72* and *GRN* carriers exhibited more pronounced alterations in M26 and M22 (0.85 to 1 difference in z-score vs. controls) than symptomatic *MAPT* (0.5 to 0.6 difference in z-score vs. controls). Presymptomatic *GRN* carriers also showed elevations in M26 spliceosome that were not present in presymptomatic *C9orf72* or *MAPT* mutation carriers. M2 presynapse and M28 synapse assembly/axon were decreased in symptomatic *GRN* and *MAPT*, with both modules exhibiting particularly strong relationships with CDR^®^+NACC-FTLD and CSF NfL in *MAPT* (M2: CDR^®^+NACC-FTLD ρ=−0.59, *p* = 1.0e-4; NfL ρ=−0.54, *p* = 8.0e-4; M28: CDR^®^+NACC-FTLD ρ=−0.43, *p* = 7.4e-3; NfL ρ=−0.46, *p* = 5.0e-3). M9 ion transport, which showed the strongest presymptomatic signal in full sample analyses, was decreased among both presymptomatic *C9orf72* and *MAPT* carriers vs. controls.

Several additional module expression patterns emerged in gene-stratified analyses that were not present when examining modules in all mutation carriers. A cluster of modules adjacent to M26 spliceosome (M24 ubiquitination/translation, M25 protein folding/metabolism, M27 metabolism) were increased and/or positively correlated with disease severity in both symptomatic *C9orf72* and *GRN*. M29 extracellular matrix (ECM), enriched for ECM proteins and microglial cell-type markers, was uniquely elevated in symptomatic *MAPT* carriers vs. controls. M4 complement/coagulation was closely related to M29 and also selectively elevated in symptomatic *MAPT* vs. controls. M3 postsynapse/glycosylation did not significantly differ between mutation carrier groups and controls, but was one of the strongest correlates of disease severity measures in *MAPT* (CDR^®^+NACC-FTLD ρ=−0.53, *p* = 7.0e-4; NfL ρ=−0.46, *p* = 5.9e-3).

### Differential Abundance Analysis.

To complement network analyses, we examined differential abundance of all 4,138 proteins to identify individual CSF proteins driving module-level differences in gene-stratified comparisons of symptomatic carriers, presymptomatic carriers, and controls (Supplementary Table 5, Extended Data Fig. 1). M26 spliceosome had the highest proportion of individual differentially abundant proteins in symptomatic *C9orf72* and *GRN* carriers (Supplementary Table 6), including nuclear proteins TRA2B, TMPO, and HNRNPAB. Proteins assigned to modules with neuronal enrichment were also differentially abundant across multiple gene groups vs. controls, including established synaptic markers NPTX2 (M2 presynapse), CNTNAP2 (M28 synapse assembly/axon), DLG4 (M3 postsynapse/glycoslyation), and 14–3-3 proteins (e.g., YWHAZ; M19 neuron migration). Symptomatic *MAPT* exhibited the highest proportion of differentially abundant proteins from these neuronal modules, particularly M28 synapse assembly/axon. M26 spliceosome had the highest proportion of differentially abundant protein members in presymptomatic *GRN* (e.g., XRCC6) and M9 ion transport had the highest proportion of differentially abundant protein members in presymptomatic *C9orf72* and *MAPT* (e.g., KCNE2, GPR6).

*GRN* mutations are characterized by progranulin protein haploinsufficiency. As expected, CSF progranulin protein (unassigned to a module) exhibited the largest decreased abundance in both pre- and symptomatic GRN carriers vs. controls. C1QTNF1 (unassigned to a module), another lysosomal protein with CSF levels shown to be strongly colinear with GRN levels^25^, exhibited a similar pattern of decreased abundance in *GRN* carriers.

### Spliceosome, extracellular matrix, and synapse associated proteins predict cognitive trajectories.

**Figure 3:**
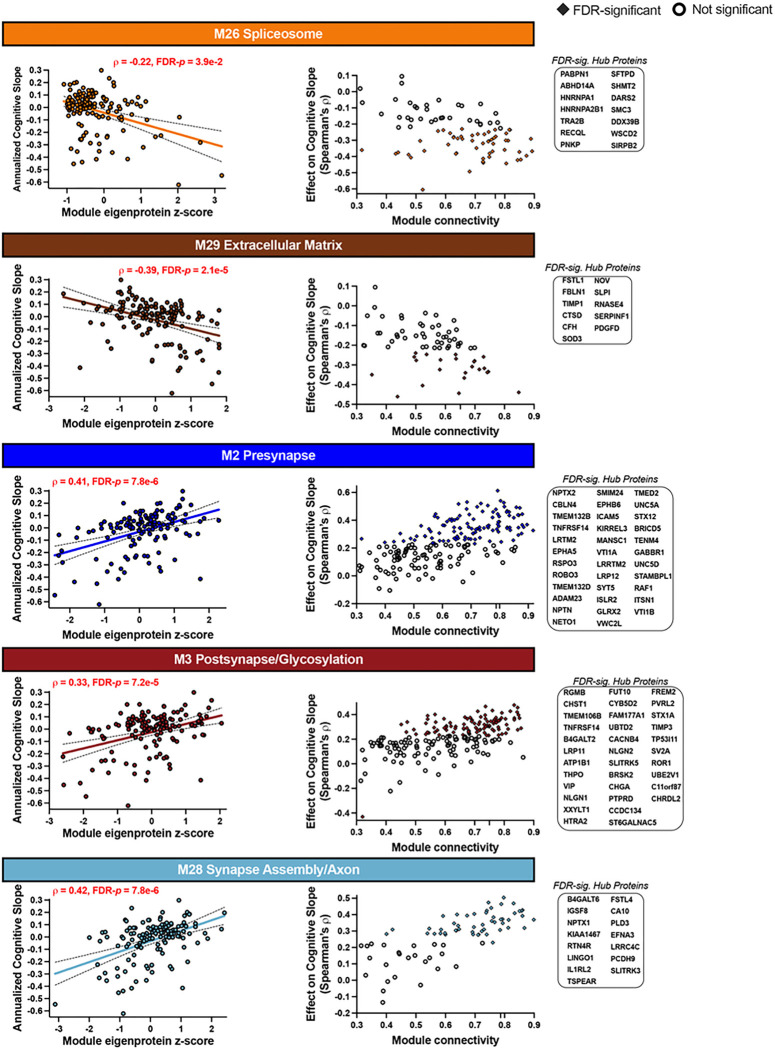
Module and Hub Protein Relationships with Cognitive Trajectory. Plots display the top 5 CSF genetic FTLD network modules most strongly associated with global cognitive trajectories in the full sample. Eigenprotein z-scores are plotted against the annual rate of global cognitive change during the study period (n = 137). Person-specific cognitive slopes were extracted from linear mixed-effects models that included baseline demographics (age, sex, education) and time (years since baseline). Confidence bands represent 95% confidence intervals for Spearman’s r values. Information on the association between all network module eigenproteins and cognitive trajectory in the full sample, within each gene, and within presymptomatic mutation carriers is provided in Supplementary Table 7. For proteins assigned to each module, an individual protein’s strength of connectivity to the module (x-axis) is plotted against the individual protein’s correlation with global cognitive change (y-axis). Proteins that exhibited stronger intramodular connectivity also exhibited stronger relationships with cognitive slope. Color-filled triangles represent individual proteins that survived false discovery rate (FDR) correction in proteome-wide differential correlational analyses (n=646 total proteins, full list in Supplementary Table 7). Proteins in the top 20^th^ percentile of intramodular connectivity and are classified as ‘hub’ proteins. Hub proteins that significantly correlated with cognitive trajectory are listed with each plot.

Module correlations with global cognitive trajectories in the full sample are provided in Supplementary Table 7 and Fig. 2A. Modules that were most strongly associated with cognitive decline in the full sample included M29 ECM (ρ=−0.39, FDR-*p* = 2.1e-5) and M26 spliceosome (ρ=−0.22, FDR-*p* = 3.9e-2). Modules that were most strongly associated with cognitive preservation included M28 synapse assembly/axon (ρ = 0.42, FDR-*p* = 7.8e-6), M2 presynapse (ρ = 0.41, FDR-*p* = 7.8e-6) and M3 postsynapse/glycoslyation (ρ = 0.33, FDR-*p* = 5.6e-4; Fig. 3).

To determine whether cognitive findings were driven by specific gene groups, post-hoc analyses examined gene-stratified correlations between modules and cognitive trajectories (Supplementary Table 7). M26 spliceosome exhibited one of the strongest associations with cognitive trajectories in *GRN*, whereas M29 ECM and synaptic/neuronal modules (M2 presyapse, M3 postsynapse/glycosylation, M28 synapse assembly/axon) were among the strongest contributors to cognitive trajectories in *C9orf72* (M29, M2, M28) and *MAPT* (M29, M2, M3). To determine the relationship between CSF modules and early stage cognitive change, analyses were also conducted within presymptomatic carriers, collapsed across gene group. In these analyses, M29 ECM (ρ=−0.44, *p* = 1.8e-3) and M2 presynapse (ρ = 0.44, p = 1.7e-3) were most strongly associated with early cognitive change.

In differential correlational analyses, 646 proteins were significantly correlated with global cognitive trajectories in the full sample (FDR-*p* < .05; Supplementary Table 6). Over half of those differentially expressed proteins were assigned to modules that also strongly associated with cognitive trajectories (M26, M29, M2, M3, M28). To determine whether individual proteins linked to cognitive trajectories were also of high influence (‘hub proteins’) in these target modules, we plotted individual protein correlations with cognitive slope against their intramodular connectivity (intramodular kME). Proteins that were module hubs, defined as top 20th percentile of intramodular kME^20^, and had FDR-corrected significance with cognitive slope are highlighted in the Fig. 3 inset protein lists. Notable ‘hub’ proteins included the neuronal pentraxins, NPTX2 (M2 presynapse; largest effect on cognitive slope across all proteins) and NPTX1 (M28 synapse assembly/axon), as well as neuroligins NLGN1 and NLGN2 (M3 postsynapse/glycoslyation). Other notable ‘hub’ proteins linked to cognitive trajectories included transmembrane proteins TMEM106B (M3 postsynapse/glycoslyation) and TMEM132B (M2 presynapse), ECM-linked proteins FSTL1 and TIMP1 (M29 ECM), and nuclear proteins HNRNPA1 and RECQL (M26 spliceosome).

### Genetic FTLD modules are preserved in sporadic PSP-RS.

**Figure 4:**
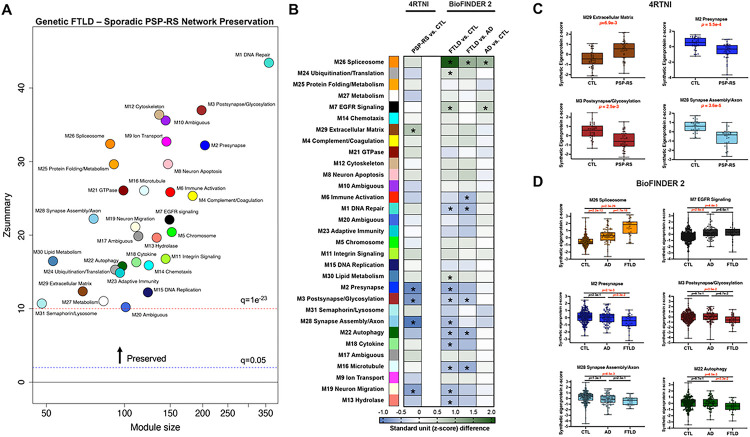
Cross-Cohort Validation. Validation cohorts included 4RTNI, composed of PSP and controls, and BioFINDER 2, composed of patients with frontotemporal dementia clinical syndromes, biomarker-confirmed Alzheimer’s disease (AD), and controls. 4RTNI CSF samples were assayed with SomaScan and BioFINDER CSF samples were assayed with Olink. **A)** Weighted gene correlational network analysis was applied to 4RTNI SomaScan data to test for module preservation across the genetic FTLD and sporadic PSP-Richardson syndrome (PSP-RS) networks. Modules that have a *Z*_summary_ score greater than or equal to 1.96 (or *q* = 0.05, blue dotted line) are considered to be preserved, and modules that have a *Z*_summary_ score greater than or equal to 10 (or *q* = 1 × 10^−23^, red dotted line) are considered to be highly preserved. All modules in the genetic FTLD network were highly preserved in the sporadic PSP-RS network. **B)** Synthetic eigenproteins were reconstructed in 4RTNI and BioFINDER 2 to test for concordance in module relationships with disease groups. Heatmap displays average synthentic eigenprotein z-score differences between PSP-RS and controls (CTL), FTLD vs. CTL, FTLD vs. AD, and AD vs. CTL. Asterisks indicate statistical significance after false discovery rate correction **C, D)** Synthetic eigenprotein boxplots for key modules from 4RTNI **(C)** and BioFINDER 2 **(D)** analyses. Pairwise differences in module synthetic eigenproteins by case status were assessed by two-sided t-test with FDR-correction. Box plots represent the median and 25th and 75th percentiles, and box hinges represent the interquartile range of the two middle quartiles within a group. Min and max data points define the extent of whiskers (error bars).

We applied the same WGCNA methods to CSF SomaScan data from an independent cohort of individuals with sporadic PSP-RS^26^ to determine whether protein co-expression patterns identified in genetic FTLD could be reproduced in sporadic FTLD, specifically a sporadic FTLD tauopathy (Supplementary Table 8B). All 31 modules from the genetic FTLD cohort were highly preserved in the sporadic PSP-RS network (all *Z*_summary_ scores > 10 [> q = 1 × 10^−23^]), indicating strong consistency of the CSF proteomic correlational architecture across genetic and sporadic disease (Fig. 4A). Next, we reconstructed the genetic FTLD network modules in PSP-RS using synthetic eigenproteins to determine whether modules derived from the genetic network could also differentiate PSP-RS from controls. Of the 31 synthetic eigenproteins derived from the genetic FTLD protein module assignments, five were significantly altered in sporadic PSP-RS vs. controls after FDR-correction (Supplementary Table 8A; Fig. 4B; decreased in PSP-RS: M28 synapse assembly/axon, M2 presynapse, M3 postsynapse/glycoslyation, M19 neuron migration; increased in PSP-RS: M29 ECM). Notably, four of these five modules were those most strongly related to *MAPT* disease severity (M2, M3, M28, M29), with consistency in directionality of effects (Fig. 4C). These results suggest that, despite differences in initial pathogenesis, genetic and sporadic forms of FTLD-tau exhibit shared proteomic signatures in CSF, characterized by decreases in neuronal cell-type specific proteins and increases in ECM proteins.

### Genetic FTLD modules differentiate FTLD from AD and controls.

To assess whether proteomic signatures from the FTLD network were specific to FTLD or more broadly reflective of neurodegeneration, irrespective of molecular etiology, we constructed synthetic eigenproteins in a second replication cohort (BioFINDER 2; Supplementary Table 9b) comprised of 29 patients with frontotemporal dementia clinical syndromes, 87 AD patients matched on demographics and disease severity, and 248 AD biomarker-negative controls. BioFINDER 2 CSF samples were analyzed using a proximity extension assay platform (Olink). Synthetic eigenproteins were computed based on protein measurements that overlapped between Olink and SomaScan, offering the opportunity to validate proteomic signatures across platforms. CSF proteomic alterations were more pronounced in FTLD than AD. Thirteen synthetic eigenproteins differed between FTLD and controls, 6 differed between FTLD and AD, and only 2 differed between AD and controls (all FDR-*p* < .05; Supplementary Table 9a; Fig. 4B). Most synthetic eigenproteins that differentiated FTLD from AD and/or controls represented modules that also differed between FTLD mutation carriers and familial controls from ALLFTD (Fig. 4D). These included increased M26 spliceosome (FTLD > AD > control) and decreased M2 presynapse (FTLD < AD, control), M28 synapse assembly/axon (FTLD, AD < control), and M22 autophagy (FTLD < AD, control). These results support the cross-platform and cross-cohort reproducibility of genetic FTLD network-derived modules, which are affected to a greater extent in FTLD than AD.

### Module Overlap with CSF AD Networks.

**Figure 5:**
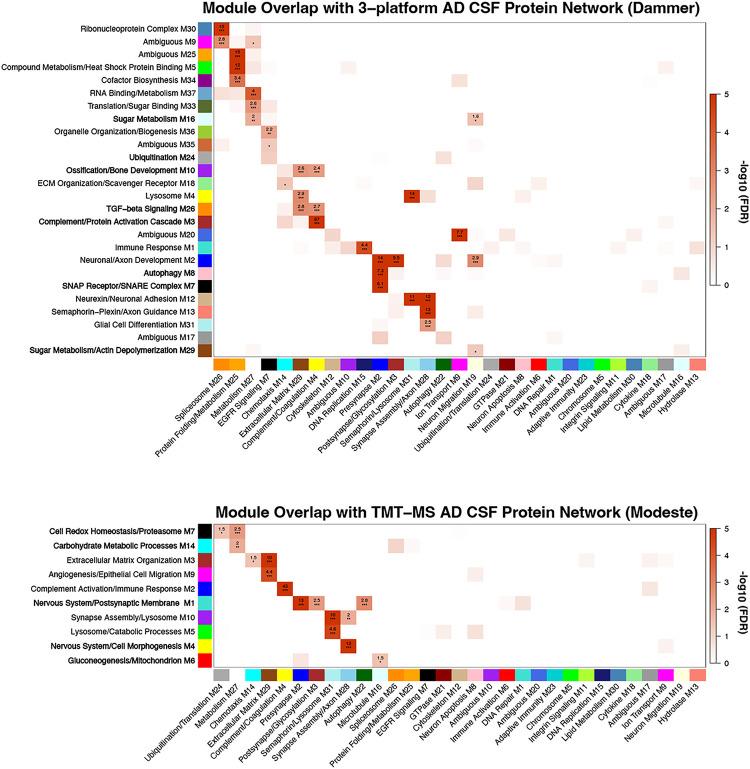
Genetic FTLD CSF Network Module Over-Representation Analysis with AD CSF Networks. Module member overrepresentation analysis (ORA) of the genetic FTLD CSF network with two CSF protein co-expression networks in Alzheimer’s disease (AD): 1) 3-platform network, obtained using SomaScan, Olink, and tandem mass tag-based mass spectrometry (TMT-MS; Dammer et al.^21^); 2) single platform network obtained using TMT-MS (Modeste et al.^27^). Box values represent −log_10_(FDR) value for pairwise module overlap, determined using one-tailed Fisher’s exact test. Bolded AD network modules significantly differed between AD and controls. Modules from the AD networks (y-axis rows) without an overlap value of − log_10_(FDR) > 1 are not included in the heatmaps.

The protein co-expression patterns and pathway enrichment present in the CSF genetic FTLD network partially resembled CSF networks previously identified in AD^21,27^. We employed module overrepresentation analyses to empirically compare the genetic FTLD network to previously constructed CSF networks in sporadic AD and thus identify CSF modules that overlap across neurodegenerative conditions. These AD networks were built using multi-platform (SomaScan + Olink + TMT-MS; Dammer et al.^21^) and TMT-MS (Modeste et. al^27^) proteomic approaches, which also allowed us to probe the influence of platform on FTLD and AD network overlap (Fig. 5). Of the 31 genetic FTLD network modules, 14 had significant overrepresentation of protein members in at least one corresponding module from the multi-platform AD network and 11 had significant overrepresentation in at least one module from the TMT-MS AD network (10 of 14 modules). M4 complement/coagulation was the most strongly overlapping FTLD network module in both AD networks, which also exhibited the strongest preservation across brain tissue, CSF and plasma in the multi-platform study^21^. M29 ECM and neuron/oligodendrocyte-enriched FTLD network modules (M2, M3, M28, M31) also exhibited strong overlap with modules from both CSF AD networks. M26 spliceosome overlapped with a module from the multi-platform network, M30 ribonucleoprotein complex, which did not differ between AD and controls. M26 spliceosome did not overlap with any module from the TMT-MS network, which broadly lacked modules with primary enrichment for RNA binding/splicing pathways. Overall, we observed strong conservation of modules linked to neuronal, oligodendrocyte, ECM, and immune processes in both FTLD and AD CSF samples.

## Discussion

We analyzed over 4,000 CSF proteins from patients across the disease severity spectrum of genetic FTLD. We observed that protein co-expression communities most strongly linked to FTLD include proteins involved in RNA processing, synaptic/axonal function, ECM, and protein degradation. These modules, and the hub proteins that drive their co-expression, correlated with cross-sectional markers of clinical severity and axonal degeneration as well as longitudinal cognitive change. We also observed CSF protein alterations in presymptomatic mutation carriers, including decreased M9 ion transport in *C9orf72* and *MAPT*, and increased M26 spliceosome in *GRN*. CSF protein signatures identified in genetic FTLD were also present in PSP-RS patients with autopsy-confirmed PSP (sporadic FTLD-tau), whose protein network alterations closely resembled the neuronal and ECM-linked proteomic signatures of *MAPT* carriers (genetic FTLD-tau). Genetic FTLD-associated modules also distinguished a cohort comprised of both sporadic and genetic frontotemporal dementia patients from controls and AD, despite cross-cohort differences in proteomic measurement platforms. Our findings support the utility of large-scale CSF proteomics in identifying biological pathways and candidate biomarkers across the spectrum of FTLD disease severity, with relevance to both genetic and sporadic forms of disease.

Spliceosomal and related nuclear proteins were markedly increased in symptomatic carriers across all three FTLD gene groups. Prior studies report increased RNA splicing protein levels across neurodegenerative disease brain tissues, including FTLD-TDP^28^ and tauopathies^29^, implicating RNA splicing dysfunction as a shared mechanism of neurodegeneration^30^. Cross-cohort replication analyses demonstrated a stair-step pattern of increased M26 spliceosome abundance across controls, AD, and FTLD. Although M26 overlapped with RNA pathway modules from the multi-platform AD network^21^, the CSF spliceosome was more strongly dysregulated in FTLD in our study than in prior studies of AD^21,27,31^. M26 was elevated prior to symptom onset and predicted cognitive decline in *GRN* carriers, highlighting the early stage prognostic utility of spliceosomal proteins in genetic FTLD-TDP. It is possible that increased CSF levels of RNA splicing proteins in FTLD-TDP may reflect compensatory or pathogenic protein network alterations in response to nuclear depletion of TDP-43 and related deficits in RNA metabolism and nucleocytoplasmic transport^32,33^. Nuclear depletion of TDP-43 increases production of HNRNP proteins^34,35^, which were also hub proteins in M26 spliceosome (e.g., HNRNPA1, HNRNPA2B1, HNRNPAB). As efforts continue to develop diagnostic biofluid markers that measure the highly-specific consequences of TDP-43 loss-of-function^36^, orthogonal development of targeted assays for proteins involved in nuclear processing may be valuable for prognosis, disease monitoring, and measuring therapeutic response.

CSF modules enriched for synaptic and other neuronal proteins, including M2 presynapse, M28 synapse assembly/axon, and M3 postysnapse/glycosylation, were strong predictors of cognitive decline. This neuronal signature of FTLD-related cognitive decline aligns with prior studies demonstrating that synaptic integrity is critical for cognitive function^37–39^. A decrease in synaptic protein abundance in FTLD, which was observed across all three study cohorts, may reflect alterations in synaptic protein turnover due to decreased protein production^40^. CSF NPTX2, previously shown to decrease in symptomatic FTLD and other neurodegenerative diseases^41–43^, exhibited the strongest association with cognitive decline out of any individual protein. Combining NPTX2 with NfL and additional synaptic markers of neurodegeneration may enhance the molecular phenotyping of neurodegeneration in FTLD and help identify patients at highest risk of cognitive progression.

Decreased abundance of M9 ion transport was a striking feature of both preysmptomatic *C9orf72* and *MAPT*. M9 was enriched for proteins that govern neuronal ion flux, including potassium voltage-gated channel proteins (e.g., KCNIP4, KCNE2) that regulate neuronal excitability and contain genetic risk loci for psychiatric disorders^44,45^. Presymptomatic alterations to M9 may reflect neuronal ion imbalances, which could contribute to neural signaling changes^46,47^ that often precede widespread neurodegeneration in FTLD^48^. Decreased M9 ion transport in presymptomatic FTLD may also reflect a longstanding neurodevelopmental molecular signature of genetic dementia, considering that M9 levels did not correlate with clinical severity. M9 hub proteins such as NNAT and FOXG1 are key regulators of early stage neuron differentiation^49,50^. Longitudinal proteomic profiling will help elucidate the timing and trajectory of changes in these neurodevelopmental proteins, which will inform their suitability as early stage FTLD biomarkers.

Similar to synaptic modules, the microglial-enriched M29 ECM predicted cognitive trajectories, even among presymptomatic mutation carriers. M29 hub proteins included ECM-secreted growth factors (e.g., FSTL1, PDGFD) as well as lysosomal and angiogenic proteins that mediate protein degradation and remodeling of the ECM (e.g., CTSD, TIMP1). In addition to providing structural support for neurons, the ECM also modulates intercellular communication and synaptic plasticity^51,52^. In AD brains, ECM proteins are positively correlated with tau pathology^20^ and ECM glycoproteins are elevated in CSF of autosomal dominant AD several decades before symptom onset^53^, suggesting that ECM dysregulation is an early event in AD pathogenesis. M29 was more strongly correlated with symptomatic *MAPT* and PSP-RS than *C9orf72* or *GRN*, which could reflect interactions between the ECM and extracellular levels of tau that are released during trans-cellular spread^54,55^.

Although our study sample is large for a CSF proteomics study in FTLD, it is smaller than CSF proteomics studies from more prevalent neurodegenerative conditions. Our analytic approach emphasized data reduction (module construction) and cross-cohort replication to identify robust biological signals within each gene group while preserving statistical power; however, an increased sample size would support more detailed subgroup comparisons focused on individual proteins, such as candidate biomarkers that discriminate genetic FTLD-TDP vs. FTLD-tau. Another limitation is that our discovery cohort proteomic search was restricted to targets captured by SomaScan, which may introduce bias in pathways identified. SomaScan has been applied in other neurodegenerative diseases^21,25,56,57^ and has high coverage for FTLD-relevant markers such as RNA splicing proteins^21^, however platforms such as Olink and TMT-MS may exhibit greater coverage for other molecular pathways (e.g., immune). Importantly, our cross-cohort/cross-platform analyses recapitulated SomaScan FTLD signatures with Olink data and demonstrated module overlap between our SomaScan only network and prior networks that included Olink and TMT-MS.

Our analysis of over 4,000 CSF proteins in FTLD, to our knowledge, reflects the largest number of proteins measured in FTLD CSF to date. Our findings highlight a diverse ensemble of CSF protein network alterations in FTLD that align with preclinical knowledge of FTLD pathophysiology. We demonstrate that FTLD CSF proteomic signatures, including those likely reflecting alterations in RNA processing and synaptic biology, are robust and reproducible across genetic and sporadic FTLD cohorts. Our integrative systems-biology approach also highlights the potential for FTLD proteomics to inform pathophysiological mechanisms that are shared and distinct across proteinopathies, including mixed tauopathies such as AD. Importantly, this FTLD CSF proteomic network provides a data anchor that can be leveraged for multi-layered analyses across matrices and ‘omics pathways, thereby offering a rich resource for FTLD and neurodegenerative biomarker research.

## Methods

### ALLFTD Cohort.

Participants included 116 carriers of pathogenic mutations in the *C9orf72* (n = 47), *GRN* (n = 32) or *MAPT* (n = 37) genes and 39 noncarrier controls from families with a known mutation in one of these genes^58^. Participants were enrolled between 2015 and 2020 in the ARTFL/LEFFTDS Longitudinal Frontotemporal Lobar Degeneration (ALLFTD) consortium (NCT04363684)^10,59^, which includes 23 collaborating centers across the US and Canada. The ALLFTD study was approved through the Trial Innovation Network at Johns Hopkins University. Local ethics committees at each of the sites approved the study, and all participants provided written informed consent or assent with proxy consent. Inclusion in the present study required completion of baseline lumbar puncture for CSF collection. Four participants were excluded from final analysis due to outlier CSF samples (see below). Clinical stage was determined with the Clinical Dementia Rating Scale (CDR^®^) plus Behavioral and Language Domains from the National Alzheimer’s Coordinating Center (NACC) FTLD module (CDR^®^+NACC-FTLD)^60–62^. Mutation carriers were classified as presymptomatic or symptomatic based on a CDR^®^+NACC-FTLD Global score of 0 (presymptomatic) or ≥0.5 (symptomatic). Clinical and demographic characteristics across the three gene groups and controls are provided in Supplementary Table 1.

### 4RTNI Cohort.

A cohort of 35 patients with a clinical diagnosis of PSP-RS were recruited through the UCSF Memory and Aging Center (MAC) and the 4-Repeat Tauopathy Neuroimaging Initiative (4RTNI; NCT01804452). Of these 35 patients, 20 went to autopsy at the UCSF MAC Neurodegenerative Disease Brain Bank and had a confirmatory primary pathological diagnosis of PSP. A comparison group of 39 community-dwelling, cognitively unimpaired controls were recruited through the UCSF Brain Aging Network for Cognitive Health (BrANCH). CSF was collected on all participants and research procedures were approved by the UCSF Institutional Review Board.

### CSF collection and processing.

CSF was collected at baseline in polypropylene tubes via lumbar puncture in lateral recumbent or sitting positions. CSF samples were centrifuged at 2000 g for 10 minutes at room temperature. Supernatant was aliquoted in 500 microliter polypropylene tubes and stored at −80°C until further analyses. All CSF samples underwent only one freeze/thaw cycle before analysis. CSF ALLFTD samples were analyzed for neurofilament light chain (NfL) using Quanterix Simoa, as previously described^4^.

### SOMAmer proteomics.

CSF samples were analyzed using a proprietary version of the SomaScan proteomics platform (SomaLogic, Boulder, CO) that captured 4,138 unique proteins. The SomaScan assay leverages slow off-rate modified aptamers (SOMAmers), which are short, single stranded deoxynucleotides that bind to protein targets with high specificity^63^. A volume of 65 microliters of CSF was used to create SOMAmer – protein reactions in 96-well plates. Tagged SOMAmer-protein complexes were captured in a bead-based assay, and levels of SOMAmer bound to sample were quantified through a fluorescent signal in DNA hybridization microarrays^64^. The reaction signal was detected digitally and expressed as aggregated Agilent relative fluorescent units (RFU), which were normalized to scale and subsequently log_2_-transformed for downstream analysis.

### Outlier removal and covariate adjustment.

Each sample had full protein data availability with no missing values. Outlier samples were identified (n = 6 [2 *C9orf72*, 1 *GRN*, and 1 control from the genetic cohort, 2 controls from the sporadic cohort]) and removed using a three-fold SD cutoff of Z-transformed sample connectivity, as previously described^20^. Nonparametric bootstrap regression was performed in controls (familial and non-familial) to obtain a median estimated coefficient from 1,000 iterations of fitting for the ffects of typical aging and sex on each protein. Median estimated coefficients were subsequently multiplied by age and sex and subtracted from participant-specific protein values to derive adjusted protein values across the entire log_2_(abundance) matrix of cases and controls in ALLFTD and 4RTNI cohorts.

### CSF protein co-expression network analysis.

In the genetic cohort, the WGCNA algorithm^20,65^ was used to generate a CSF protein network from the n = 4,138 log_2_ protein abundance × *n* = 155 case–sample matrix that had undergone covariate correction and network connectivity outlier removal as described above. The WGCNA blockwiseModules function was run with the following parameters: power = 12, deepSplit = 4, minModuleSize = 30, mergeCutHeight = 0.07, TOMdenom=“mean”, bicor correlation, signed network type, PAM staging and PAM respects dendro as TRUE, with clustering completed within a single block. Module memberships were then iteratively reassigned to enforce kME table consistency, as previously described^20^. This module membership reassignment procedure increased the smallest module size in the network from 34 to 48 (M31) and reduced the gray (unassigned) protein count for the network from 832 (20.1%) to 199 (4.8%).

### Gene ontology and cell type enrichment.

To characterize protein module biology, we retrieved gene ontology annotations from the Bader Lab’s monthly updated .GMT formatted ontology lists downloaded October 2, 2023^66^. A Fisher’s exact test for enrichment was performed for each module’s protein membership with the background proteome consisting of all proteins measured in the current study. Pruned output with overrepresentation z-scores was visualized using an R script (https://github.com/edammer/GOparallel). Modules were also tested for cell type-specific enrichment with Fisher’s exact one-tailed tests that compared module protein members with an in-house human cell type marker list^20^.

### Differential abundance analysis.

Differentially abundant proteins were identified by one-way ANOVA, followed by Tukey’s post hoc correction for pairwise comparisons between symptomatic carriers, presymptomatic carriers, and controls (stratified analysis for each gene). Volcano plots were made using an R script that color-coded individual data points by CSF network module membership (https://github.com/edammer/parANOVA).

### Cognitive trajectory analysis.

ALLFTD participants completed an annual comprehensive neuropsychological battery, covering episodic memory, executive functions, and language skills (average [range] visits = 3.4 [1–7])^10,67^. Raw test scores were converted to *z*-scores based on the score distribution in the larger cognitively unimpaired BrANCH cohort^68^, which were then averaged into domain-based composite scores. Domain-based composite scores were averaged together to create a global cognitive composite score. The rate of cognitive change over time for each participant was determined via a linear mixed-effects model that examined global cognitive scores as a function of time (years since baseline), entered as a fixed and random effect, adjusting for baseline age, sex and years of education. Person-specific random slopes were extracted to represent the annual rate of cognitive change per participant, which was the primary outcome in downstream analyses that examined differential correlation of modules and individual proteins with cognitive trajectory.

### BioFINDER 2 Cohort.

To test whether FTLD CSF proteomic signatures were robust to platform and cohorts, we leveraged CSF proteomic data analyzed on the Olink platform in the Swedish BioFINDER 2 cohort (NCT03174938)^69^. This BioFINDER 2 replication cohort included 29 patients with frontotemporal dementia clinical syndromes, 87 AD-biomarker positive patients with MCI or dementia, and 248 AD-biomarker negative controls^70^. Of the 29 FTLD patients, 10 had genetically confirmed FTLD (8 *C9orf72*, 1 *GRN*, 1 *MAPT*) with a behavioral variant frontotemporal dementia (bvFTD) clinical syndrome. The remaining 19 cases included 13 bvFTD and 6 semantic variant primary progressive aphasia. AD cases were matched to FTLD spectrum cases 3-to-1 for age, sex, disease severity, and mean protein level^69^ using k-nearest neighbors. BioFINDER 2 participants were recruited at Skåne University Hospital and the Hospital of Ängelholm, Sweden, diagnosed by multidisciplinary assessment after clinical and neuropsychological examination, brain MRI, and lumbar puncture. The study was approved by the Regional Ethics Committee in Lund, Sweden. All participants gave written informed consent to participate.

### Validation analysis: Module preservation.

A sporadic PSP-RS (4RTNI) protein co-expression network (4,138 log_2_ protein abundance × 74 case–sample matrix) was constructed using the same blockwiseModules parameters and module reassignment procedures as described above. Preservation between the genetic FTLD and sporadic PSP-RS CSF networks was performed using the WGCNA modulePreservation function with 500 permutations. Z_summary_ composite preservation scores for 8 underlying network parameters were obtained using the mutation network as the template and PSP-RS network as the target^20,71^. Statistical significance of module preservation (minus log10 [FDR-adjusted *p* values]) was visualized as a function of module size.

### Validation analysis: Synthetic modules.

Network validation was also assessed in the 4RTNI and BioFINDER 2 cohorts by calculating synthetic eigenproteins^20,22^, which captures the variance of all protein module members present in the target cohorts. We leveraged every protein module membership assignment from the genetic FTLD network to construct synthetic eigenproteins in sporadic PSP-RS, given that CSF from both cohorts were analyzed on the same platform. Synthetic eigenprotein calculations in the BioFINDER 2 Olink dataset were restricted to Olink proteins that overlapped with SomaScan proteins from the genetic FTLD network and had missing frequency < 75% (856 total proteins). Boxplots visualized synthetic eigenprotein differences across cases and controls in each target cohort.

### Validation analysis: Module overrepresentation.

To determine the degree to which CSF modules in the genetic FTLD network overlapped with CSF modules in AD, we performed over-representation analysis (ORA) of module gene symbols between the CSF genetic FTLD network and two CSF AD protein co-expression networks that differed in depth of proteomic coverage and sample size. Specifically, the CSF genetic FTLD network was compared to a 1) deep, three-platform CSF network (Somalogic, Olink, tandem mass tag-based mass spectrometry [TMT-MS]; n = 7,158 proteins, 38 modules) constructed in a small cohort of AD (n = 18) and controls (n = 18); and 2) a shallower TMT-MS CSF network (n = 1,840 proteins, 14 modules) constructed in a larger and more racially diverse (~ 50% African American) cohort of AD (n = 98) and controls (n = 105). Module overlap was determined using one-tailed Fisher’s exact test, followed by correction of p-values for multiple testing using the Benjamini-Hochberg method. Overlap of module gene symbols between networks was visualized using a custom in-house script.

### Other statistics.

Statistical analyses were performed in R (v4.3.1). Boxplots represent the median, 25th, and 75th percentile extremes; thus, hinges of a box represent the interquartile range of the two middle quartiles of data within a group. Minimum and maximum data points define the extent of whiskers (error bars). Correlations were performed using Spearman’s rho (ρ) coefficients. Comparisons between two groups were performed by a two-sided t test. Comparisons among three or more groups were performed with ANOVA with Tukey’s pairwise comparison of significance. *P* values were adjusted for multiple comparisons by FDR correction according to the Benjamini-Hochberg method where indicated.

## References

[1] Bang J, Spina S, Miller BL (2015) Frontotemporal dementia. Lancet 386:1672–1682 26595641 10.1016/S0140-6736(15)00461-4 PMC5970949

[2] Knopman DS, Roberts RO (2011) Estimating the number of persons with frontotemporal lobar degeneration in the US population. J Mol Neurosci 45:330–335 21584654 10.1007/s12031-011-9538-y PMC3208074

[3] Mackenzie IRA, Neumann M (2016) Molecular neuropathology of frontotemporal dementia: insights into disease mechanisms from postmortem studies. J Neurochem 138:54–70 27306735 10.1111/jnc.13588

[4] Rojas JC (2021) Plasma Neurofilament Light for Prediction of Disease Progression in Familial Frontotemporal Lobar Degeneration. Neurology 96:e2296–e2312 33827960 10.1212/WNL.0000000000011848 PMC8166434

[5] Rosen HJ, Boeve BF, Boxer AL (2020) Tracking disease progression in familial and sporadic frontotemporal lobar degeneration: Recent findings from ARTFL and LEFFTDS. Alzheimers Dement 16:71–78 31914219 10.1002/alz.12004 PMC6953606

[6] Del Campo M (2022) New developments of biofluid-based biomarkers for routine diagnosis and disease trajectories in frontotemporal dementia. Alzheimers Dement 18:2292–2307 35235699 10.1002/alz.12643 PMC9790674

[7] Gifford A, Praschan N, Newhouse A, Chemali Z (2023) Biomarkers in frontotemporal dementia: Current landscape and future directions. Biomarkers Neuropsychiatry 8:100065

[8] Boxer AL (2019) New directions in clinical trials for frontotemporal lobar degeneration: Methods and outcome measures. Alzheimer’s Dement 10.1016/j.jalz.2019.06.4956 PMC6949386 31668596

[9] Greaves CV, Rohrer JD (2019) An update on genetic frontotemporal dementia. J Neurol 266:2075–2086 31119452 10.1007/s00415-019-09363-4 PMC6647117

[10] Staffaroni AM (2022) Temporal order of clinical and biomarker changes in familial frontotemporal dementia. Nat Med 28:2194–2206 36138153 10.1038/s41591-022-01942-9 PMC9951811

[11] Ferrari R, Manzoni C, Hardy J (2019) Genetics and molecular mechanisms of frontotemporal lobar degeneration: an update and future avenues. Neurobiol Aging 78:98–110 30925302 10.1016/j.neurobiolaging.2019.02.006

[12] Balendra R, Isaacs AM (2018) C9orf72-mediated ALS and FTD: multiple pathways to disease. Nat Reviews Neurol 14:544–558 10.1038/s41582-018-0047-2 PMC6417666 30120348

[13] Cook CN (2020) C9orf72 poly(GR) aggregation induces TDP-43 proteinopathy. Sci Transl Med 12 10.1126/scitranslmed.abb3774 PMC7989020 32878979

[14] Kao AW, McKay A, Singh PP, Brunet A, Huang EJ (2017) Progranulin, lysosomal regulation and neurodegenerative disease. Nat Rev Neurosci 18:325–333 28435163 10.1038/nrn.2017.36 PMC6040832

[15] Kumar-Singh S, Progranulin (2011) TDP-43: mechanistic links and future directions. J Mol Neurosci 45:561–573 21863317 10.1007/s12031-011-9625-0 PMC3207122

[16] Strang KH, Golde TE, Giasson BI (2019) MAPT mutations, tauopathy, and mechanisms of neurodegeneration. Lab Invest 99:912–928 30742061 10.1038/s41374-019-0197-x PMC7289372

[17] Schmidt S, Holzer M, Arendt T, Sonntag M, Morawski M (2022) Tau Protein Modulates Perineuronal Extracellular Matrix Expression in the TauP301L-acan Mouse Model. Biomolecules 12 10.3390/biom12040505 PMC9027016 35454094

[18] Tracy TE (2022) Tau interactome maps synaptic and mitochondrial processes associated with neurodegeneration. Cell 185:712–728e714 35063084 10.1016/j.cell.2021.12.041 PMC8857049

[19] Rayaprolu S (2021) Systems-based proteomics to resolve the biology of Alzheimer’s disease beyond amyloid and tau. Neuropsychopharmacology 46:98–115 32898852 10.1038/s41386-020-00840-3 PMC7689445

[20] Johnson ECB (2022) Large-scale deep multi-layer analysis of Alzheimer’s disease brain reveals strong proteomic disease-related changes not observed at the RNA level. Nat Neurosci 25:213–225 35115731 10.1038/s41593-021-00999-y PMC8825285

[21] Dammer EB (2022) Multi-platform proteomic analysis of Alzheimer’s disease cerebrospinal fluid and plasma reveals network biomarkers associated with proteostasis and the matrisome. Alzheimers Res Ther 14:174 36384809 10.1186/s13195-022-01113-5 PMC9670630

[22] Johnson ECB (2020) Large-scale proteomic analysis of Alzheimer’s disease brain and cerebrospinal fluid reveals early changes in energy metabolism associated with microglia and astrocyte activation. Nat Med 26:769–780 32284590 10.1038/s41591-020-0815-6 PMC7405761

[23] Gendron TF (2022) Comprehensive cross-sectional and longitudinal analyses of plasma neurofilament light across FTD spectrum disorders. Cell Rep Med 3:100607 35492244 10.1016/j.xcrm.2022.100607 PMC9044101

[24] Delaby C (2020) Differential levels of Neurofilament Light protein in cerebrospinal fluid in patients with a wide range of neurodegenerative disorders. Sci Rep 10:9161 32514050 10.1038/s41598-020-66090-x PMC7280194

[25] Phillips B (2023) Proteome wide association studies of LRRK2 variants identify novel causal and druggable proteins for Parkinson’s disease. npj Parkinson’s Disease 9:107 10.1038/s41531-023-00555-4 PMC10329646 37422510

[26] Coyle-Gilchrist IT (2016) Prevalence, characteristics, and survival of frontotemporal lobar degeneration syndromes. Neurology 86:1736–1743 27037234 10.1212/WNL.0000000000002638 PMC4854589

[27] Modeste ES (2023) Quantitative proteomics of cerebrospinal fluid from African Americans and Caucasians reveals shared and divergent changes in Alzheimer’s disease. Mol Neurodegener 18:48 37468915 10.1186/s13024-023-00638-z PMC10355042

[28] Umoh ME (2018) A proteomic network approach across the ALS-FTD disease spectrum resolves clinical phenotypes and genetic vulnerability in human brain. EMBO Mol Med 10:48–62 29191947 10.15252/emmm.201708202 PMC5760858

[29] Johnson ECB (2018) Deep proteomic network analysis of Alzheimer’s disease brain reveals alterations in RNA binding proteins and RNA splicing associated with disease. Mol Neurodegener 13:52 30286791 10.1186/s13024-018-0282-4 PMC6172707

[30] Nikom D, Zheng S (2023) Alternative splicing in neurodegenerative disease and the promise of RNA therapies. Nat Rev Neurosci 24:457–473 37336982 10.1038/s41583-023-00717-6

[31] Higginbotham L (2020) Integrated proteomics reveals brain-based cerebrospinal fluid biomarkers in asymptomatic and symptomatic Alzheimer’s disease. Sci Adv 6 10.1126/sciadv.aaz9360 PMC7577712 33087358

[32] Liu EY (2019) Loss of Nuclear TDP-43 Is Associated with Decondensation of LINE Retrotransposons. Cell Rep 27:1409–1421e1406 31042469 10.1016/j.celrep.2019.04.003 PMC6508629

[33] Hofmann JW, Seeley WW, Huang EJ (2019) RNA Binding Proteins and the Pathogenesis of Frontotemporal Lobar Degeneration. Annu Rev Pathol 14:469–495 30355151 10.1146/annurev-pathmechdis-012418-012955 PMC6731550

[34] Bampton A, Gittings LM, Fratta P, Lashley T, Gatt A (2020) The role of hnRNPs in frontotemporal dementia and amyotrophic lateral sclerosis. Acta Neuropathol 140:599–623 32748079 10.1007/s00401-020-02203-0 PMC7547044

[35] Deshaies J-E (2018) TDP-43 regulates the alternative splicing of hnRNP A1 to yield an aggregation-prone variant in amyotrophic lateral sclerosis. Brain 141:1320–1333 29562314 10.1093/brain/awy062 PMC5917749

[36] Irwin KE (2023), A fluid biomarker reveals loss of TDP-43 splicing repression in pre-symptomatic ALS. bioRxiv

[37] Casaletto KB (2017) Neurogranin, a synaptic protein, is associated with memory independent of Alzheimer biomarkers. Neurology 89:1782–1788 28939668 10.1212/WNL.0000000000004569 PMC5664306

[38] Wingo AP (2019) Large-scale proteomic analysis of human brain identifies proteins associated with cognitive trajectory in advanced age. Nat Commun 10:1619 30962425 10.1038/s41467-019-09613-z PMC6453881

[39] Camporesi E (2020) Fluid Biomarkers for Synaptic Dysfunction and Loss. Biomark Insights 15:1177271920950319 32913390 10.1177/1177271920950319 PMC7444114

[40] Paterson RW (2019) SILK studies - capturing the turnover of proteins linked to neurodegenerative diseases. Nat Rev Neurol 15:419–427 31222062 10.1038/s41582-019-0222-0 PMC6876864

[41] Boiten WA (2020) Pathologically Decreased CSF Levels of Synaptic Marker NPTX2 in DLB Are Correlated with Levels of Alpha-Synuclein and VGF. Cells 10 10.3390/cells10010038 PMC7824459 33383752

[42] Libiger O (2021) Longitudinal CSF proteomics identifies NPTX2 as a prognostic biomarker of Alzheimer’s disease. Alzheimers Dement 17:1976–1987 33984181 10.1002/alz.12353 PMC9222372

[43] van der Ende EL (2020) Neuronal pentraxin 2: a synapse-derived CSF biomarker in genetic frontotemporal dementia. J Neurol Neurosurg Psychiatry 91:612–621 32273328 10.1136/jnnp-2019-322493 PMC7279197

[44] Weißflog L (2013) KCNIP4 as a candidate gene for personality disorders and adult ADHD. Eur Neuropsychopharmacol 23:436–447 22981920 10.1016/j.euroneuro.2012.07.017

[45] Tam GW (2010) Confirmed rare copy number variants implicate novel genes in schizophrenia. Biochem Soc Trans 38:445–451 20298200 10.1042/BST0380445

[46] Zhang L (2023) Network Connectivity Alterations across the MAPT Mutation Clinical Spectrum. Ann Neurol 94:632–646 37431188 10.1002/ana.26738 PMC10727479

[47] Lee SE (2017) Network degeneration and dysfunction in presymptomatic C9ORF72 expansion carriers. Neuroimage Clin 14:286–297 28337409 10.1016/j.nicl.2016.12.006 PMC5349617

[48] Huber N (2022) Deficient neurotransmitter systems and synaptic function in frontotemporal lobar degeneration—Insights into disease mechanisms and current therapeutic approaches. Mol Psychiatry 27:1300–1309 34799692 10.1038/s41380-021-01384-8 PMC9095474

[49] Hou PS, hAilín D, Vogel T, Hanashima C (2020) Transcription and Beyond: Delineating FOXG1 Function in Cortical Development and Disorders. Front Cell Neurosci 14:35 32158381 10.3389/fncel.2020.00035 PMC7052011

[50] Pitale PM, Howse W, Gorbatyuk M (2017) Neuronatin Protein in Health and Disease. J Cell Physiol 232:477–481 27442611 10.1002/jcp.25498

[51] Pintér P, Alpár A (2022) The Role of Extracellular Matrix in Human Neurodegenerative Diseases. Int J Mol Sci 23 10.3390/ijms231911085 PMC9569603 36232390

[52] De Luca C, Colangelo AM, Virtuoso A, Alberghina L, Papa M, Neurons (2020) Glia, Extracellular Matrix and Neurovascular Unit: A Systems Biology Approach to the Complexity of Synaptic Plasticity in Health and Disease. Int J Mol Sci 21 10.3390/ijms21041539 PMC7073232 32102370

[53] Johnson ECB (2023) Cerebrospinal fluid proteomics define the natural history of autosomal dominant Alzheimer’s disease. Nat Med 29:1979–1988 37550416 10.1038/s41591-023-02476-4 PMC10427428

[54] Moretto E, Stuart S, Surana S, Vargas JNS, Schiavo G (2022) The role of extracellular matrix components in the spreading of pathological protein aggregates. Front Cell Neurosci 16:844211 35573838 10.3389/fncel.2022.844211 PMC9100790

[55] Holmes BB, Diamond MI (2014) Prion-like properties of Tau protein: the importance of extracellular Tau as a therapeutic target. J Biol Chem 289:19855–19861 24860099 10.1074/jbc.R114.549295 PMC4106306

[56] Shi L (2019) Discovery and validation of plasma proteomic biomarkers relating to brain amyloid burden by SOMAscan assay. Alzheimer’s Dement 15:1478–1488 31495601 10.1016/j.jalz.2019.06.4951 PMC6880298

[57] Yang C (2021) Genomic atlas of the proteome from brain, CSF and plasma prioritizes proteins implicated in neurological disorders. Nat Neurosci 24:1302–1312 34239129 10.1038/s41593-021-00886-6 PMC8521603

[58] Ramos EM (2020) Genetic screening of a large series of North American sporadic and familial frontotemporal dementia cases. Alzheimers Dement 16:118–130 31914217 10.1002/alz.12011 PMC7199807

[59] Boeve B (2020) The longitudinal evaluation of familial frontotemporal dementia subjects protocol: Framework and methodology. Alzheimer’s Dement 16:22–36 31636026 10.1016/j.jalz.2019.06.4947 PMC6949411

[60] Knopman DS, Weintraub S, Pankratz VS (2011) Language and behavior domains enhance the value of the clinical dementia rating scale. Alzheimer’s Dement 7:293–299 21575870 10.1016/j.jalz.2010.12.006 PMC3096831

[61] Knopman DS (2008) Development of methodology for conducting clinical trials in frontotemporal lobar degeneration. Brain 131:2957–2968 18829698 10.1093/brain/awn234 PMC2725027

[62] Miyagawa T (2020) Utility of the global CDR(^®^) plus NACC FTLD rating and development of scoring rules: Data from the ARTFL/LEFFTDS Consortium. Alzheimers Dement 16:106–117 31914218 10.1002/alz.12033 PMC7202045

[63] Rohloff JC (2014) Nucleic Acid Ligands With Protein-like Side Chains: Modified Aptamers and Their Use as Diagnostic and Therapeutic Agents. Mol Ther Nucleic Acids 3:e201 25291143 10.1038/mtna.2014.49 PMC4217074

[64] Gold L (2010) Aptamer-based multiplexed proteomic technology for biomarker discovery. PLoS ONE 5:e15004 21165148 10.1371/journal.pone.0015004 PMC3000457

[65] Langfelder P, Horvath S (2008) WGCNA: an R package for weighted correlation network analysis. BMC Bioinformatics 9:559 19114008 10.1186/1471-2105-9-559 PMC2631488

[66] Reimand J (2019) Pathway enrichment analysis and visualization of omics data using g:Profiler, GSEA, Cytoscape and EnrichmentMap. Nat Protoc 14:482–517 30664679 10.1038/s41596-018-0103-9 PMC6607905

[67] Weintraub S (2018) Version 3 of the Alzheimer Disease Centers’ Neuropsychological Test Battery in the Uniform Data Set (UDS). Alzheimer Dis Assoc Disord 32:10–17 29240561 10.1097/WAD.0000000000000223 PMC5821520

[68] Saloner R (2024) Plasma phosphorylated tau-217 exhibits sex-specific prognostication of cognitive decline and brain atrophy in cognitively unimpaired adults. Alzheimer’s Dement 20:376–387 37639492 10.1002/alz.13454 PMC10843677

[69] Linda K (2023), Cerebrospinal fluid reference proteins increase accuracy and interpretability of biomarkers for brain diseases. bioRxiv, 2023.2006.2008.544222 10.1038/s41467-024-47971-5 PMC11063138 38693142

[70] Santillo AF (2023) [18F]RO948 tau positron emission tomography in genetic and sporadic frontotemporal dementia syndromes. Eur J Nucl Med Mol Imaging 50:1371–1383 36513817 10.1007/s00259-022-06065-4 PMC10027632

[71] Langfelder P, Luo R, Oldham MC, Horvath S (2011) Is my network module preserved and reproducible? PLoS Comput Biol 7:e1001057 21283776 10.1371/journal.pcbi.1001057 PMC3024255

